# Pro-apoptotic and anti-angiogenic actions of 2-methoxyestradiol and docosahexaenoic acid, the biologically derived active compounds from flaxseed diet, in preventing ovarian cancer

**DOI:** 10.1186/s13048-019-0523-3

**Published:** 2019-05-25

**Authors:** Purab Pal, Karen Hales, Jim Petrik, Dale Buchanan Hales

**Affiliations:** 1 Department of Physiology, Southern Illinois University, 1125 Lincoln Drive, Life Science II, Room 245B, Carbondale, IL 62901 USA; 2 Department of Obstetrics and Gynecology, Southern Illinois University School of Medicine, Springfield, IL 62702 USA; 3 Department of Biomedical Sciences, Ontario Veterinary College, University of Guelph, Guelph, ON N1G 2W1 Canada

**Keywords:** Ovarian cancer, Flaxseed, 2-methoxyestradiol, Docosahexaenoic acid, p38-MAPK, Apoptosis, Angiogenesis

## Abstract

**Background:**

We have previously shown that a whole flaxseed supplemented diet decreased the onset and severity of ovarian cancer in the laying hen, the only known animal model of spontaneous ovarian cancer. Flaxseed is rich in omega-3 fatty acids (OM3FA), mostly α-Linoleic acid (ALA), which gets converted to Docosahexaenoic acid (DHA) by the action of delta-6 desaturase enzyme. Ingestion of flaxseed also causes an increase in production of 2-methoxyestradiol (2MeOE_2_) via the induction of the CYP1A1 pathway of estrogen metabolism. We have previously reported that the flaxseed diet induces apoptosis via p38-MAPK pathway in chicken tumors. The objective of this study was to investigate the effect of the flaxseed diet on ovarian cancer in chickens, focusing on two hallmarks of cancer, apoptosis and angiogenesis.

**Results:**

The anti-cancer effects of two active biologically derived compounds of flax diet, 2MeOE_2_ and DHA, were individually tested on human ovarian cancer cells and in vivo by the Chick Chorioallantoic Membrane (CAM) assay. Our results indicate that a flaxseed-supplemented diet promotes apoptosis and inhibits angiogenesis in chicken tumors but not in normal ovaries. 2MeOE_2_ promotes apoptosis in human ovarian cancer cells, inhibits angiogenesis on CAM and its actions are dependent on the p38-MAPK pathway. DHA does not have any pro-apoptotic effect on human ovarian cancer cells but has strong anti-angiogenic effects as seen on CAM, but not dependent on the p38-MAPK pathway.

**Conclusions:**

Dietary flaxseed supplementation promotes a pro-apoptotic and anti-angiogenic effect in ovarian tumors, not in normal ovaries. The biologically derived active compounds from flaxseed diet act through different pathways to elicit their respective anti-cancer effects. A flaxseed-supplemented diet is a promising approach for prevention of ovarian cancer as well as having a significant potential as an adjuvant treatment to supplement chemotherapeutic agents for treatment of advanced stages of ovarian cancer.

**Electronic supplementary material:**

The online version of this article (10.1186/s13048-019-0523-3) contains supplementary material, which is available to authorized users.

## Background

Ovarian cancer is one of the deadliest gynecological cancers, ranking fifth in all cancer-related deaths in women. Unavailability of suitable predictive biomarkers make the disease hard to detect until stage III/IV leading to its poor prognosis and fewer treatment options. Consequently, the 5-year survival rate for all epithelial types of ovarian cancer in the United States is currently 47%; the estimated number of ovarian cancer cases to be diagnosed in 2018 is 22,240 and the number of estimated deaths is 14,070 in United States, per SEER cancer statistics [1].

The laying hen provides the only animal model that develops the disease naturally. Histologically, the disease closely resembles the human form of the disease [2–4]. Expression of different molecular markers [5–8], symptoms such as profuse ascitic fluid and peritoneal metastasis in stage III/IV of the disease [7, 9], are found to be very similar to the human disease. Ovarian cancer is also directly correlated to the number of ovulations during the lifespan in both women and hens. A woman potentially ovulates around 400 times prior to menopause, and the average age of diagnosis is 63. Chickens start laying eggs approximately by the age of 5 months, and thus ovulate around 400 times by their 2nd year of lay [2]. By two and a half years, a significant number of hens will have developed ovarian cancer. Reducing the number of ovulations in hens [10, 11] has been shown to decrease the incidence of ovarian cancer, a phenomenon also observed in women.

Our research using the chicken model has shown that a flaxseed diet can reduce both the severity and incidence of ovarian cancer [9, 12]. Flaxseed is a rich source of omega-3 fatty acids (OM3FA), predominantly α-Linoleic acid (ALA), the phytoestrogen lignan secoisolaricisresinol diglucoside (SDG), and several other macronutrients, fiber and minerals [13]. The current study was designed to investigate the effects of the OM3FA and phytoestrogens separately. ALA is converted to eicosapentaenoic acid (EPA) and docosahexaenoic acid (DHA) and SDG is converted to enterodiol (ED) and enterolactone (EL). DHA has been shown to have anti-inflammatory and cardioprotective properties while ED and EL are known to have anti-estrogenic and antioxidant properties [14, 15]. EL serum levels have been monitored in post-menopausal women diagnosed with breast cancer and who have received flaxseed supplements. The increase in EL in serum and disease-free survival has been positively correlated [16].

Estradiol is metabolized in the liver through its hydroxylation by three different cytochrome P450 (CYP) enzymes. CYP1A1 produces the 2-hydroxy metabolites, while CYP1B1 and CYP3A4 yield 4-hydroxy and 16-hydroxy metabolites, respectively [17]. 2-hydroxy estrogens can be readily converted into 2-methoxy [18] estrogens by catechol-O-methyltransferase enzyme (COMT) [19]. 2-methoxyestradiol (2MeOE_2_) is easily excreted and known to be the least potent estrogenic metabolite while the 4-hydroxyestradiol is readily oxidized to a genotoxic compound, 3,4 quinone. Therefore, the whole flax diet favors the CYP1A1 pathway for generation of 2MeOE_2_, resulting in a higher 2-hydroxy:16-hydroxy estradiol ratio, which has been shown to be protective against postmenopausal breast cancer [20]. 

From our previous studies, we have shown that the flax diet promotes the CYP1A1 pathway of estrogen metabolism while decreasing both CYP1B1 and CYP3A4 in the pre-neoplastic chicken ovaries. The upregulation in CYP1A1 enzyme also parallels the increase in 2-hydroxy: 16-hydroxy estradiol ratio and the 2MeOE_2_ level in the serum of chickens [21]. 2MeOE_2_ is an established anti-proliferative and anti-apoptotic agent [22, 23] and has been tested on different cancer cell lines over the past few years [24–30]. Previously, we have shown that a whole flax diet promotes apoptosis in the chicken ovaries and also activation of the p38-MAPK pathway [31].

The objective of this study was to explore mechanisms of the anti-cancer effects of the flaxseed diet in ovarian carcinogenesis and explore how the biologically active components of flaxseed diets, 2MeOE_2_ and DHA, accomplish their actions, focusing on two hallmarks of cancer, apoptosis and angiogenesis.

## Results

### Flax diet induces apoptosis in ovarian tumors in chicken

**Fig. 1:**
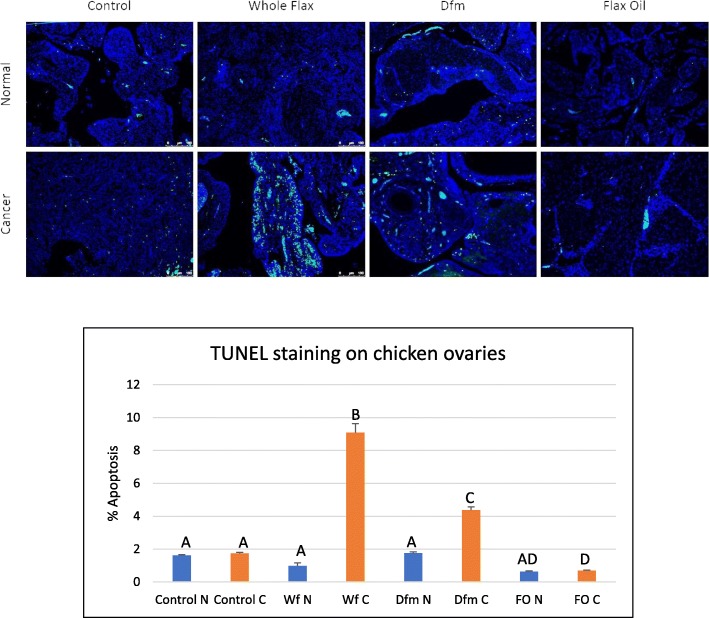
TUNEL staining on normal and cancerous chicken ovarian tissue TUNEL staining on normal and cancerous chicken ovarian tissue from control, whole flax, defatted flax meal and flax oil diet groups. Two-way ANOVA, error bars: SD, *p* < 0.05

Hens were on four different diets (see Table 1 for their respective compositions) for eleven months, following which tumors and normal ovaries were collected, fixed embedded and sectioned. TUNEL staining was performed to determine if diet had an effect on the extent of apoptosis in the tumors. TUNEL-positive cells were found to be significantly increased in tumors from whole flaxseed-fed chickens compared to normal ovaries (Fig. 1). The defatted flax meal (DFM) diet also caused an increase in TUNEL positive cells in tumors compared to normal ovaries, though to a lesser extent than in whole flaxseed-fed chickens. The number of TUNEL positive cells in all normal ovaries and tumors from control and flax oil-fed hens were not significantly different.

### Flax diet inhibits angiogenesis in ovarian tumors in chicken

**Fig. 2:**
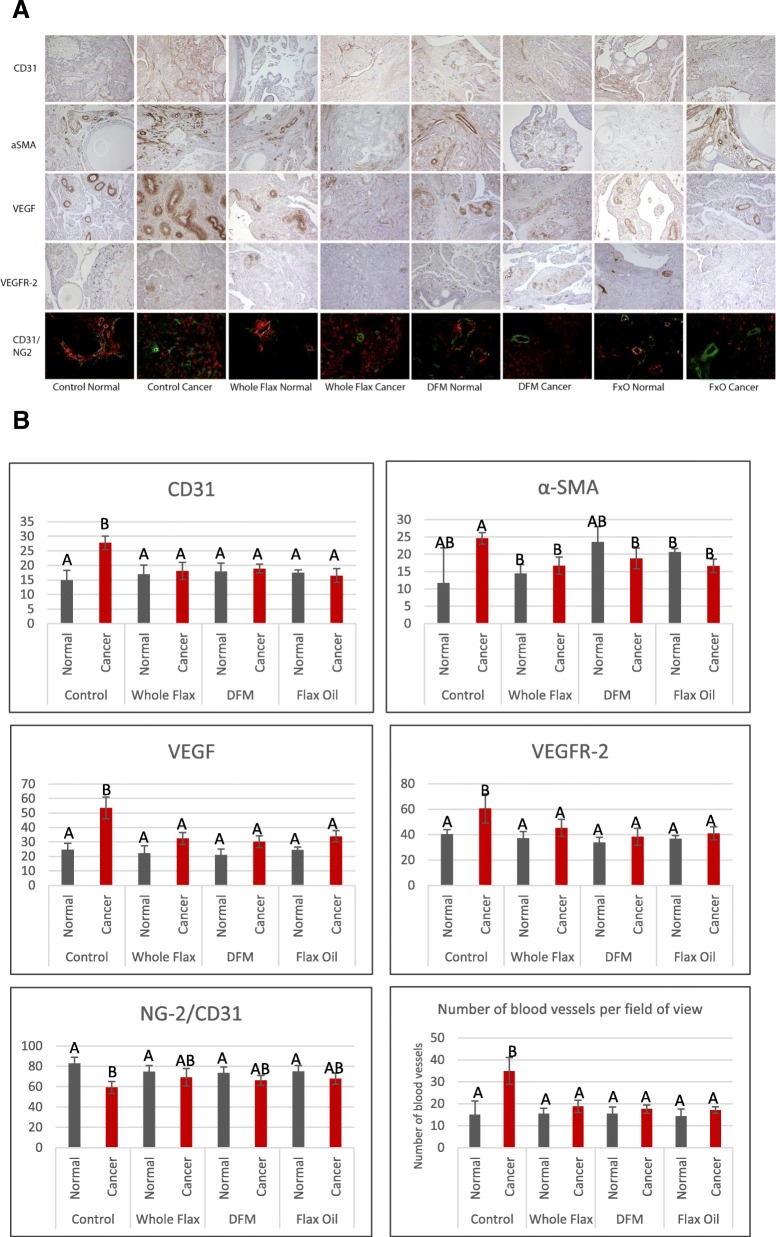
Immmunohistochemical staining on normal and cancerous chicken ovarian tissue for angiogenic markers **a** Normal and cancerous chicken ovaries from control, whole flax, defatted flax meal and flax oil groups were stained with anti-CD31, anti-⍺ smooth muscle actin, anti-vascular endothelial growth factor, anti-vascular endothelial growth factor receptor type 2 antibodies and NG2 co-stained with CD31. **b** Quantified expression of the angiogenic markers, number of positive cells over total number of cells per field of view. Two-way ANOVA, error bars: SD, *p* < 0.05

Following the feeding trial, tumors and normal ovaries were stained for CD31, an endothelial cell marker, α-smooth muscle actin, a marker of perivascular smooth muscle cells, vascular endothelial growth factor (VEGF), and vascular endothelial growth factor receptor (VEGFR-2), to determine if there is an effect of diet on angiogenesis. CD31 immunostaining showed increased endothelial cells in chicken ovarian tumors with the control diet. All three flax diets reduced CD31 staining in chicken ovarian tumors with no significant changes in the normal ovaries. α-SMA expression in the normal chicken ovaries had substantial variability, and no significant difference was observed in the flax groups compared to control diet in normal ovaries. However, all three flax groups had reduced α-SMA in ovarian tumors compared to the control fed hens. Tumors and normal ovaries were also stained with NG-2, a specific marker for pericytes. A significant reduction in pericyte to endothelial cell ratio (NG-2 co-staining with CD31) was observed in ovarian tumors compared to normal ovaries in control fed hens. No significant changes in the ratio of pericyte to endothelial cell were observed across different diets, either in normal ovaries, or in tumors. All three flax diets also reduced expression VEGF and VEGFR-2 in ovarian tumors with no change observed in normal ovaries (Fig. 2) or ovarian tumors from control fed hens. The number of blood vessels per field of view was counted for each group and all three flax diets showed significant decrease in the number of blood vessels in ovarian tumors with no changes observed in normal ovaries (Fig. 2).

### 2MeOE_2_ induces apoptosis in human ovarian cancer cells

**Fig. 3:**
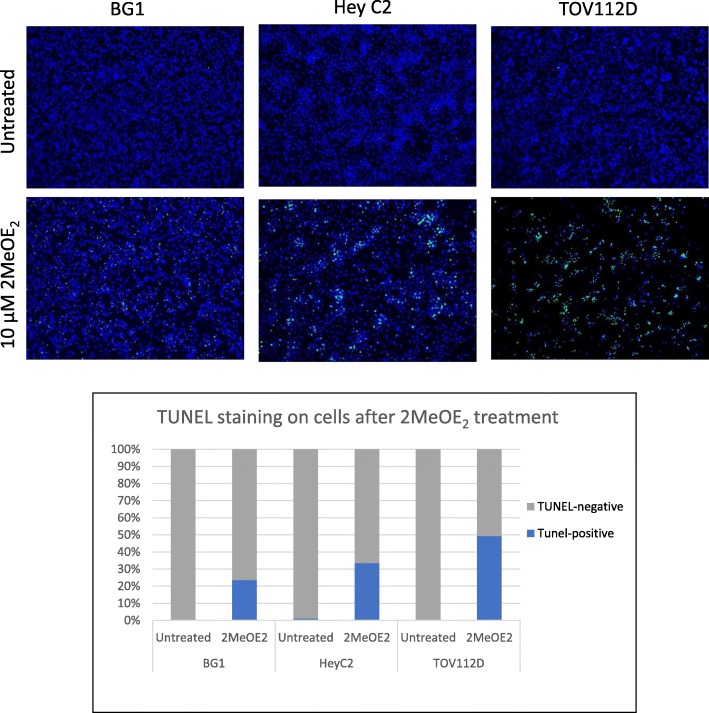
TUNEL staining on BG1, HeyC2 and TOV112D cells after 2MeOE_2_ treatment BG1, HeyC2 and TOV112D cells treated with 10 μM 2MeOE_2_ and after an incubation of 26 h TUNEL staining was performed

BG1, HeyC2 and TOV112D cells grown on coverslips were treated with a 10 μM 2MeOE_2_ and incubated for 26 h. The number of TUNEL-positive cells was increased significantly in all three cell lines, compared to their respective controls (Fig. 3). BG1 cells had a 23.6% increase in TUNEL positive cells compared to the untreated control. HeyC2 and TOV112D cells had 33.5 and 49.4% increase in TUNEL positive cells compared to their respective controls after 2MeOE_2_ treatment.

### Action of 2MeOE_2_ is not dependent on estrogen receptor α (ERα) expression

BG1, HeyC2 and TOV112D cells were seeded on coverslips and stained with an anti-ERα (Rabbit polyclonal) antibody. BG1 cells were found to be positive for ERα while the other two cell lines do not express ESR1 (ERα) (Additional file 1: Figure S1).

### 2MeOE_2_ exerts its anti-apoptotic effects via the p38-MAPK pathway

**Fig. 4:**
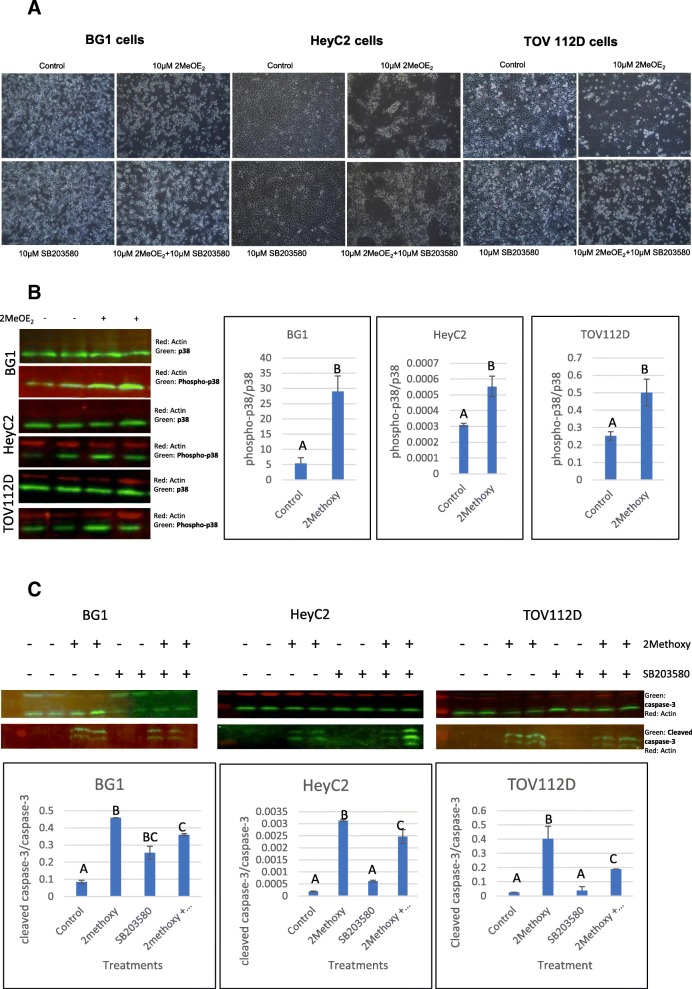
2MeOE_2_ treatment of human ovarian cancer cells **a** BG1, HeyC2 and TOV112D cells were treated with 10 μM 2MeOE_2_, 10 μM SB203580 and 10 μM 2MeOE_2_ and SB203580 together. Cells were photographed after 24 h from treatment. 10 μM 2MeOE_2_ plates had significant reduction in cell numbers from the control plate. **b** Western blot analysis performed on protein lysates from control and 10 μM 2MeOE_2_ against p38 and phospho-p38 on BG1, HeyC2 and TOV112D cells (*n* = at least 3 for each dataset). One-way ANOVA, error bars: SEM, *p* < 0.05. **c** Western blot analysis performed on protein lysates from control, 10 μM 2MeOE_2_, 10 μM SB203580 and 10 μM 2MeOE_2_ + 10 μM 2MeOE_2_ against caspase 3 and cleaved caspase 3 on BG1, HeyC2 and TOV112D cells (*n* = 3). Amount of caspase-3 cleaved into the active cleaved caspase-3 was quantified to estimate the amount of apoptosis induced by each treatment. Two-way ANOVA, error bars: SEM, *p* < 0.05

BG1, HeyC2 and TOV112D cells were treated with a control media, +/− 10 μM 2MeOE_2_ and 10 μM SB203580, a selective p38 MAPK inhibitor. At the 24-h time point, 2MeOE_2_ treated cells were fewer in number compared to the control group for all three cell lines. Cells appeared more rounded up compared to the control. Cells treated with 10 μM SB203580 were not visibly different compared to the control group. The group treated with both 2MeOE_2_ and SB203580 had greater viability compared to the 10 μM 2MeOE_2_ group. (Fig. 4a). Western blots demonstrated that the amount of phosphorylated p38-MAPK/total p38-MAPK was significantly increased after the 10 μM 2MeOE_2_ treatment compared to the control group (Fig. 4b). To correlate the association of p38-MAPK activation with induction of apoptosis, western blots were also done for cleaved caspase-3 and total caspase-3. The cleaved caspase-3/total caspase-3 ratio was significantly increased after 2MeOE_2_ and was reduced by the addition of SB203580 (Fig. 4c).

### 2MeOE_2_ reduces viability in human ovarian cancer cells

The effect of 2MeOE_2_ on the viability of the ovarian carcinoma cells was tested by the MTS assay. 10 μM 2MeOE_2_ significantly reduced the viability in all three cell lines (Additional file 1: Figure S2). The decrease in viability is significantly altered after the cells were treated with 10 μM SB203580. 10 μM SB203580 alone did not alter the viability significantly in any of the cell lines compared to the respective controls.

### DHA does not induce apoptosis nor p38-MAPK activation in ovarian cancer cells

**Fig. 5:**
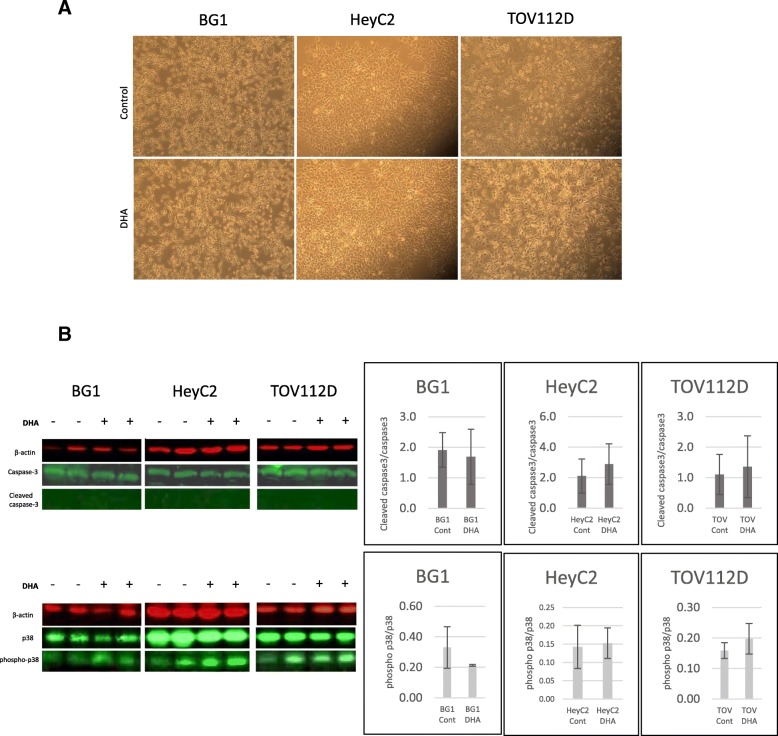
DHA treatment of human ovarian cancer cells **a** BG1, HeyC2 and TOV112D cells were treated with 10 μM DHA. Cells were photographed after 24 h of treatment. **b** Western blot from whole cell lysates following 10 μM DHA treatment for p38, phospho-p38, caspase-3 and cleaved caspase-3 (*n* = at least 3 for each experiment)

BG1, HeyC2 and TOV112D cells were treated with 10 μM DHA. Following a 24-h incubation, cells appeared to look normal and there was no visible reduction in cell number (Fig. 5a). Western blot from whole cell lysates showed no significant alteration in phosphorylated p38/total p38 expression after DHA treatment, nor was there any significant cleavage of caspase-3 (Fig. 5b).

### Both 2MeOE_2_ and DHA inhibit angiogenesis in a dose-dependent manner in CAM

**Fig. 6:**
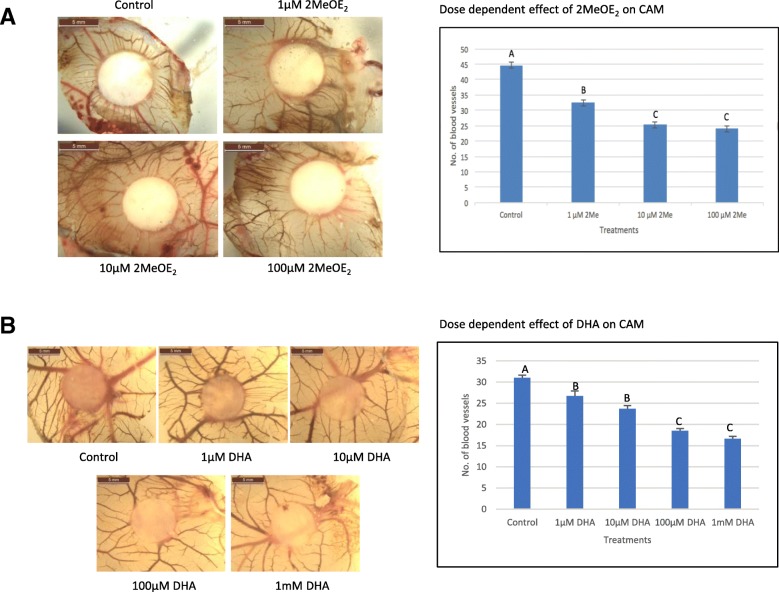
Anti-angiogenic effect of 2MeOE_2_ and DHA on CAM **a** 2MeOE_2_ was added to a filter paper in increasing concentrations of 1 μM (*n* = 9), 10 μM (*n* = 5) and 100 μM (*n* = 6), and placed on the CAM. Control group (*n* = 5) was treated with only the solvent, 100% ethanol. After an incubation of 48 h, CAMs were harvested and viewed under the microscope and number of sprouting vessels around the filter paper was counted. One-way ANOVA, error bars: SEM, *p* < 0.05. **b** DHA was added to a filter paper in increasing concentration – 1 μM (*n* = 9), 10 μM (*n* = 5), 100 μM (*n* = 6) and 1 mM (*n* = 8); and placed on the CAM. Control group (*n* = 10) treated with only the solvent, 100% ethanol. Following an incubation of 48 h, CAMs were harvested and viewed under the microscope and number of sprouting vessels around the filter paper was counted. One-way ANOVA, error bars: SEM, *p* < 0.05

Doses of 1 μM, 10 μM and 100 μM 2MeOE_2_ were applied to CAM to investigate its role on angiogenesis. The number of vessels was significantly decreased in 1 μM 2MeOE_2_ treated groups compared to the control. Angiogenesis was significantly inhibited in a dose-dependent manner up to 10 μM 2MeOE_2_, with no further reduction in angiogenesis beyond this concentration. CAMs from the 10 μM and 100 μM 2MeOE_2_ treated groups showed a zone of vascularization inhibition around the filter paper (Fig. 6a). A similar dose-dependent anti-angiogenic response was observed upon treatment with increasing concentrations of DHA (1 μM, 10 μM, 100 μM and 1 mM) compared to the control (Fig. 6b).

### Anti-angiogenic effect of 2MeOE_2_ is p38-MAPK dependent, but DHA is p38-MAPK independent

**Fig. 7:**
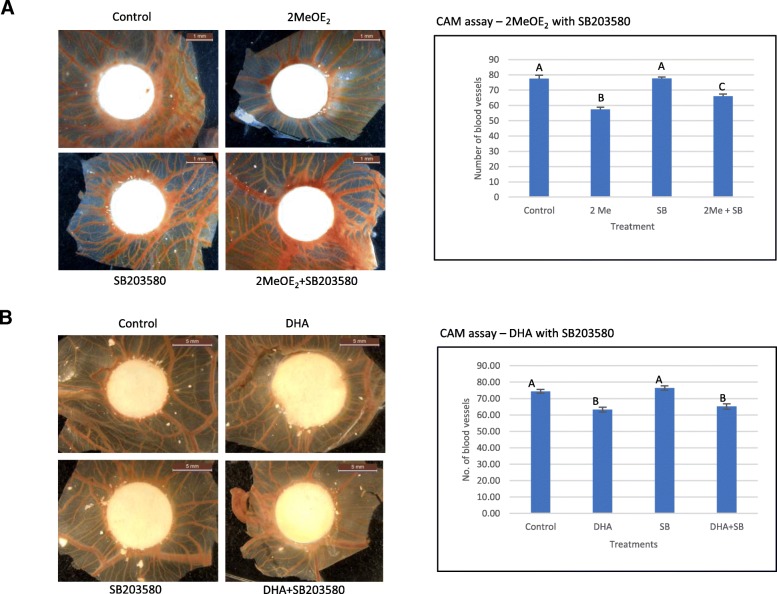
Involvement of p38-MAPK pathway in anti-angiogenic action of 2MeOE_2_ and DHA on CAM **a** 10 μM 2MeOE_2_ (*n* = 10), 10 μM SB203580 (*n* = 5) or both 2MeOE_2_ and SB203580 were added to filter papers and placed on CAMs. Control group (*n* = 6) was treated with only the solvent, 100% ethanol. Two-way ANOVA, error bars: SEM, *p* < 0.05. **b** 10 μM DHA (*n* = 8), 10 μM SB203580 (*n* = 8) or both DHA and SB203580 together (*n* = 11) were added to filter papers and placed on CAMs. Control group (*n* = 9) was treated with only the solvent, 100% ethanol. Two-way ANOVA, error bars: SEM, *p* < 0.05

To examine whether the anti-angiogenic effects of 2MeOE_2_ and DHA were mediated via the p38-MAPK pathway, the CAM assay was performed with 10 μM SB203580 combined with 10 μM 2MeOE_2_ or 10 μM DHA. Addition of SB203580 affected the inhibitory action of 2MeOE_2_ (Fig. 7a) but had no effect on the anti-angiogenic action of DHA (Fig. 7b). No significant effect on angiogenesis occurred with 10 μM SB203580 alone.

## Discussion

The purpose of this study was to investigate the mechanisms through which the biologically active components of flaxseed drive apoptosis and inhibit tumor angiogenesis. The present study demonstrated that 2MeOE_2_ promotes apoptosis and inhibits angiogenesis in vitro and both the actions are dependent on the p38-MAPK pathway. DHA has an anti-angiogenic effect but does not induce apoptosis in ovarian cancer cells, and its effects are not p38-MAPK dependent. The effects of 2MeOE_2_ and DHA were independent of ER-α expression in human ovarian cancer cells.

Previously we have shown that a whole flaxseed diet induces apoptosis in chicken ovarian tumors, promotes phosphorylation of p38-MAPK [31] and alters both estrogen signaling and metabolism in chickens. Flax diets induce CYP1A1 expression resulting in an increase in 2MeOE_2_ levels in serum [31]. The OM3FA (predominantly ALA, which gets systemically converted to DHA) components of the flaxseed diet have been previously described as having potent anti-inflammatory effects in cancerous chicken ovaries by reducing prostaglandin E_2_ (PGE_2_) levels as well as PTGS-2 (COX-2) gene expression [12]. Taken together, these observations suggested that different components of flaxseed play different roles in the anti-cancer mechanisms of flaxseed and work in a synergistic way in the whole flaxseed diet. This study was designed to individually investigate the mechanisms of action of 2MeOE_2_ and DHA, which are two principal biologically-derived compounds from the flaxseed diet, in inducing apoptosis and inhibiting angiogenesis in ovarian cancer.

Previously we have shown that a 15% flaxseed diet induces apoptosis in chicken tumors but not in normal chicken ovaries [31]. Here we investigated the effects of the lignan vs oil fractions of flax seed. The DFM diet induced apoptosis in chicken tumors but not in normal chicken ovaries. The flax oil diet did not cause a significant increase in apoptosis in normal or cancerous chicken ovaries. However, whole flax diet had a much greater pro-apoptotic effect than the DFM diet on chicken ovarian tumors, suggesting an anti-cancer entourage effect provided by the whole flaxseed-supplemented diet where the pro-apoptotic effect of the whole seed exceeds the sum of the effects of the individual components.

Immunostaining for various angiogenic markers suggested that the whole flaxseed diet exerts an anti-angiogenic effect in chicken ovarian tumors. The number of blood vessels per field of view significantly increased in ovarian tumors compared to normal ovaries in control fed hens. All three flaxseed diets have caused a significant decrease in the number of vessels in ovarian tumors with no significant changes in the normal ovaries. CD31 immunostaining across different groups showed that whole flaxseed diet significantly reduced the endothelial cell population in ovarian tumors, but had no effect on angiogenesis in normal ovarian tissues in hens. α-SMA immunostaining indicated the α-SMA positive cells decreased in ovarian tumors, as previously reported in one of our studies [32]. α-SMA is expressed in the pericytes, cells that divide and increase in number to surround and pack the newly formed vessels for vascular stabilization. Additionally, α-SMA is also expressed in cancer-associated fibroblasts (CAFs) and other smooth muscle cells The decrease of α-SMA in ovarian tumors indicates a reduction in the fibrotic condition in the ovaries of flaxseed-fed hens. One of the classic characteristics of ovarian cancer is smooth muscle metaplasia and excessive proliferation in ovarian fibroblasts resulting in a fibrotic condition [33]. This phenomenon, known as desmoplasia, contributes to the protection of the tumor cells and facilitates tumor growth by physically shielding them from the immune cells [34]. The fibrosity of the intra-tumoral stroma and the adjacent normal tissues also induces a high inflammatory burden, and diminishes the response to anti-cancer treatments. The whole flaxseed supplemented diet mediates the reduction in the fibrosity of the ovarian tumor suggesting that dietary supplementation with flaxseed could enhance the therapeutic efficacy of the treatment regimen.

However, the non-specific expression of α-SMA limited our ability to assess the maturity of the tumor vasculature. It is important to remember that tumor angiogenesis is distinctly different from growth and maturation of normal blood vessels. The hypoxic tumor micro-environment drives the growth and proliferation of the endothelial cells but the growth also renders an increase in the ratio of endothelial cells to pericytes. This results in immature, dysfunctional and leaky vessel formation. In order to evaluate the maturity of tumor angiogenesis, we immunostained the normal and cancerous ovaries with NG-2, a specific pericyte marker [35] Quantification of total number of CD31 and NG-2 positive cells per field of view indicated a decrease in pericyte to endothelial cell ratio in tumors in the control diet. The flax diets increased the ratio, indicating an improvement of the vasculature and decrease in leakiness (*p* > 0.05). Vascular endothelial growth factor (VEGF) is one of the most potent angiogenic factors expressed as a response to tissue hypoxia, and known to most effectively bind to its receptor (VEGFR-2/Flk-1) in endothelial cells promoting endothelial cell proliferation. Both VEGF and VEGFR-2 expressions significantly increased in cancer from control diet-fed chickens and decreased in cancer from chickens fed the whole flaxseed diet, with no change in normal ovarian tissues.

One of our recent works has suggested that a recombinant thrombospondin-1 type 1 repeats (3TSR) pre-treatment normalizes advanced ovarian tumor vasculature and improves the uptake of chemotherapeutic drugs [36]. Minimizing the toxic treatments as well as increasing chemotherapeutic efficacy is a desired goal in clinical oncology. Our findings in this study demonstrate that a flaxseed supplemented diet causes a reduction in angiogenesis in chicken ovarian tumors, with no significant changes in normal ovaries, thereby normalizing the vasculature of the ovarian tumors. Thus, flaxseed supplementation could be a promising combinational approach for the treatment of advanced stage ovarian carcinoma in addition to its use as a preventive therapy.

The role of ERα in the prognosis of ovarian carcinoma is controversial. Some studies have shown a correlation between ERα expression and improved outcomes in epithelial ovarian carcinoma patients [37, 38], whereas other studies show increased ERα expression decreases the overall survival of ovarian cancer patients [39, 40]. Several studies suggest that the onset of estrogen-mediated cell proliferation and cell survival leading to ovarian carcinogenesis is mediated through increased ERα expression [41, 42]. However, ERα expression also varies between different types of ovarian cancer [43]. In our previous studies, we have shown that a 15% whole flax diet significantly reduces the ERα expression in pre-neoplastic chicken ovaries [31]. Expression of ESR-2 in the chicken ovaries is very low [44] and ovarian cancer has been reported to have a higher ESR1/ESR2 ratio in both chicken and women [45–47]. We have tested the anti-cancer actions of 2MeOE_2_ on both ERα-positive (BG1) and ERα-negative (HeyC2 and TOV112D) cells and found that 2MeOE_2_ exerted similar effects, regardless of ER-status. These results suggest that the anti-cancer actions of 2MeOE_2_ are ER-independent.

MAPK14, or p38α, was the first identified member of the MAPK family. p38-MAPK is phosphorylated on threonine and tyrosine residues in response to stress [48]. Activation of p38-MAPK has been linked to the phosphorylation of the pro-apoptotic protein BAX, facilitating its translocation to the Mitochondrial Outer Membrane (MOM) and inducing apoptosis [49]. Phosphorylated p38-MAPK also activates a group of transcription factors such as CHOP, ATF1, p53, MEF2C and MEF2A [50, 51] that are potential mediators in the apoptotic pathway. In the current study, cells treated with 2MeOE_2_ showed significant changes in viability and morphology which was partially reversed by the p38 MAPK inhibitor (SB203580) [52]. Western blots showed that the 2MeOE_2_ treatment induces activation of p38-MAPK in all three cell lines and cleavage of caspase-3 paralleled phosphorylation of p38-MAPK. The cleavage of caspase-3 was significantly reduced after the cells were treated with 2MeOE_2_ and SB203580, suggesting the p38-MAPK pathway contributes to the pro-apoptotic activity of 2MeOE_2_. The 2MeOE_2_ mediated reduction in viability of the human ovarian cancer cells was also reversed by SB203580. The in vivo angiogenesis assay showed that 2MeOE_2_ exhibits an anti-angiogenic effect in a dose-dependent manner on CAM. These results agree with our previous observations that a whole flax diet increases the phospho-p38 expression in chicken ovaries and promotes apoptosis in chicken ovarian tumors [31].

Flaxseed is also one of the richest sources of OM3FA, mostly ALA. ALA is converted into longer-chain EPA and DHA. The conversion efficiency of this pathway in humans varies in terms of ethnicity and dietary habits. However, several groups have shown that a flaxseed diet increases this conversion efficiency in mammals [53]. EPA has been suggested to act as a competitive inhibitor of arachidonic acid that binds to COX-2 [54] and previously we have reported that dietary OM3FA reduces PGE_2_ level in the serum and COX-2 gene (PTGS-2) expression in laying hens [12]. The effect of DHA alone on apoptosis and angiogenesis was tested in the human ovarian cancer cells. DHA has been reported to bind the peroxisome proliferator-activated receptor (PPAR) α and γ, as well as IκB, which sequesters NFκB in the cytosol, thus preventing transcription of a myriad of pro-inflammatory NFκB response genes [55]. In the current study, DHA was found not to have any pro-apoptotic effect on human ovarian cancer cells. Following a 24-h incubation, DHA treatment did not induce any caspase-3 cleavage, nor did it activate p38-MAPK which indicates that DHA effects are not mediated by p38-MAPK.

We have investigated the individual effects of 2MeOE_2_ and DHA on angiogenesis by CAM assay. The chorioallantois in the chick embryo is formed in the first 4 to 5 days of embryonic development. This is when the outer mesodermal layers of the allantois and the chorion fuse together forming a connection of blood vessels that grows rapidly in the next few days. Judah Folkman first described that this window of time can be utilized for the study of angiogenesis [56]. Since then, CAM has been extensively used as an assay to study the angiogenic effects of various test compounds with different treatment methods such as filter disks, gelatin sponge, collagen implants or other gelated materials. In the present study, we have performed a filter disk based CAM assay. Both 2MeOE_2_ and DHA reduced the number of sprouting vessels around the filter paper, demonstrating an anti-angiogenic effect. The effect of 2MeOE_2_ is reduced by treatment with SB203580, suggesting p38-MAPK pathway plays a role in promoting the anti-angiogenic effects of 2MeOE_2_. At the same time, SB203580 had no effect on the anti-angiogenic action of DHA suggesting that DHA exerts its anti-angiogenic effects in a p38-MAPK independent way.

Although the canonical signaling of p38-MAPK pathway is believed to promote proliferation and invasiveness in different cancers across different studies, there have been recent studies that have shown MAP kinases to be involved in promoting apoptosis in tumor cells. 2MeOE_2_ is known to bind at or near the colchicine-binding site of the tubulin, suppressing microtubule dynamics, and thus eliciting mitotic arrest [57] in tumors both in vitro and in vivo. Recent studies with 2MeOE_2_ treatment have shown that it induces JNK, Erk-1/2 and p38 activation in breast cancer cells [58], stabilizes SMAD7 and induces p38-MAPK mediated apoptosis in prostate cancer [59] and retinoblastoma cells [49]. In our previous work, we have shown that the flax diet activates p38-MAPK in chicken ovarian tumors and promotes apoptosis [31]. The current study suggests that the pro-apoptotic and anti-angiogenic effects of 2MeOE_2_ are dependent on p38-MAPK, however the anti-angiogenic effect of DHA is not dependent on p38-MAPK pathway.

2-MeOE_2_ has been clinically tested as an administered drug in ovarian cancer [60] and multiple myeloma patients [61], yielding promising results. We have shown that consumption of whole flaxseed in the diet promotes endogenous production of 2MeOE_2_, a pro-apoptotic and anti-angiogenic compound that reduces the ovarian tumor burden, and can provide an alternative natural therapeutic modality. Flaxseed diets also provide the essential, anti-inflammatory polyunsaturated fat, DHA. Consumption of flaxseed is being tested as maintenance therapy to prevent recurrence of ovarian cancer in women {https://clinicaltrials.gov/ct2/show/NCT02324439}. Adding flaxseed to the daily diet may be an important preventative measure in healthy women.

## Conclusions

A flaxseed-supplemented diet, which increases the systemic production of 2MeOE_2_ and DHA, induces apoptosis and decreases angiogenesis in ovarian tumors but not in normal ovarian tissues. 2MeOE_2_ and DHA both have anti-angiogenic effects. 2MeOE_2_ has pro-apoptotic effects but not DHA. Anti-cancer actions of 2MeOE_2_ are dependent on p38-MAPK pathway, however the actions of DHA do not involve p38-MAPK. Dietary supplementation with flaxseed may help prevent ovarian cancer in women or help them live with ovarian cancer instead of die from it.

## Methods

### Materials

The HeyC2 cell line [62] was obtained from Dr. Jean Hurteau at Northshore University Health-Evanston Hospital; TOV112D (CRL11731) cell line was purchased from ATCC. BG1 cells were obtained from Dr. Ken Korach’s lab at NIEHS [63, 64]. HyClone DMEM culture media (with and without phenol red) from ThermoFisher (SH30604.02); 2-methoxyestradiol from Sigma-Aldrich (M6383); SB203580 p38-MAPK inhibitor from Cayman chemical (13067); DHA (3687) from Tocris Biosciences; 100x HALT protease and phosphatase inhibitor cocktail from ThermoFisher (78440); DyLight™800 conjugated goat anti-rabbit IgG antibody (H&L) (35571) and DyLight™680 conjugated goat anti-rabbit IgG antibody (H&L) (35518) from Thermofisher. Alexa-594 donkey anti-rabbit secondary (133200) from Jackson Immuno Research; DeadEnd Fluorometric TUNEL system kit (G3250) and CellTitre 96® Aqueous one solution cell proliferation assay kit (G3582) were both purchased from Promega (Madison, WI, USA).

### Animal study and tissue collection

Two and half year-old White leghorn Hy-line W-36 chickens (*Gallus domesticus*) were fed either control diet (*n* = 175), diet supplemented with whole flax seed (*n* = 160), defatted flax meal (*n* = 160) or flax oil (*n* = 160) for 11 months (Table 1). All animals were housed in the animal care facility on a 17 h/7 h light/dark cycle at the University of Illinois in Urbana-Champaign. Animal care and diet protocols were approved by the Institutional Animal Care and Use Committees (IACUC) at both Southern Illinois University, Carbondale and University of Illinois at Urbana-Champaign. Birds were sequentially bled throughout the study by wing vein puncture and at the end of the study, they were euthanized by CO_2_ asphyxiation, necropsied, ovaries and liver tissues collected. Small yellow follicles (6–8 mm) and pre-ovulatory follicles (9–35 mm) were excluded. Ovaries were confirmed to have cancer by histology. Collected ovaries were dissected into ~ 2 mm pieces and either flash frozen in liquid nitrogen and stored at − 80 °C or fixed in neutral buffered formalin (NBF) fixative solution and paraffin embedded for histological staining.

**Table 1:** Diet composition

Diet	Control	15% whole flaxseed	10% defatted flax meal	5% flax oil
Enriched in	–	ALA+SDG	SDG	ALA
Ingredients (%)				
Corn	67.40	47.58	54.90	52.00
Flaxseed (whole)	0.00	15.00	0.00	0.00
Soy bean meal	18.30	18.30	18.30	18.30
Corn gluten meal	3.00	0.00	0.00	5.00
Flax oil	0.00	0.00	0.00	5.00
Defatted flax meal	0.00	0.00	10.00	0.00
Qual fal	0.00	2.50	3.80	0.00
Solka floc	0.30	5.62	1.99	8.70
Limestone	8.75	8.75	8.75	8.75
Dical	1.50	1.50	1.50	1.50
Salt	0.30	0.30	0.30	0.30
Vitamin mix	0.20	0.20	0.20	0.20
Mineral mix	0.15	0.15	0.15	0.15
DL-met	0.10	0.10	0.10	0.10
Calculated analysis				
CP, %	16.56	16.50	17.04	16.40
TME, kcal/kg	2816.00	2815.00	2816.00	2815.00
Calcium, %	3.73	3.75	3.77	3.73
Phosphorus, %	0.38	0.38	0.40	0.37
Met + Cys, %	0.67	0.64	0.72	0.67

### Immunohistochemistry

Formalin fixed ovarian tissue was paraffin embedded, 5 μM sections were cut and mounted on SuperFrost plus microscopic slides. Following deparaffinization and rehydration, slides were treated with 3% (v/v) hydrogen peroxide for 10 mins to inhibit endogenous peroxide activities. Tissues were then blocked in 5% bovine serum albumin with 0.02% sodium azide for 10 min and incubated with primary antibodies overnight at 4°c. Following 1x PBS rinse, biotinylated secondary antibodies from respective species were applied for a 2-h incubation at room temperature (1:100 dilution, Sigma) with horseradish peroxidase (Extravidin, 1:50 dilution, Sigma) followed by a brief incubation with diaminobenzidine tetrahydrochloride (Sigma). Tissues were counterstained with Carazzi’s hematoxylin for 1 min, dehydrated and mounted with Permount (Sigma).

For immunofluorescence, after rehydration and blocking with 5% BSA, sections were simultaneously stained overnight with anti-CD31 and anti-NG-2. Sections were stained with secondary antibodies against anti-CD31 (Alexa Fluor®594 nm, red, 1:100) and α-SMA (Alexa Fluor®488 nm, green, 1:100) for 1 h at room temperature. Images obtained under both 594 nm and 488 nm channels using a Fluorescent microscope (Olympus) and Metamorph Imaging software (Burlingame CA).

### Cell culture and treatments

BG1, HeyC2 and TOV112D cells were cultured in DMEM (with phenol-red) media supplemented with 10% fetal bovine serum and 7500 IU penicillin, 7500 μg streptomycin, incubated at 5% CO_2_ and 37 °C. Cells were seeded with a density of 4X10^5^ cells per well in 6-well tissue culture plates. Media was changed after 24 h to phenol-red free DMEM supplemented with 10% charcoal stripped newborn calf serum and 0.75% of 10,000 μg/ml penicillin-streptomycin. 2MeOE_2_, DHA and SB203580 (stock in DMSO) all were prepared in the phenol-red free DMEM media and added to the cells. Following a 24-h incubation, cells were photographed, harvested and total protein was extracted.

### Protein isolation from cells

Cells were scraped off after adding 200 μl ice-cold PBS and collected in Eppendorf tubes. Tubes were centrifuged at 2000 g at 4 °C for 3 min, supernatant discarded and the pellets were resuspended in 30 μl of protein lysis buffer (1x HALT protease and phosphatase inhibitor cocktail in 0.1% SDS/1xPBS). Following a short burst of sonication, protein quantities were estimated by BCA method and stored at − 20°c.

### Western blot analysis

Western blot was performed as described previously [65]. 30 μg of total protein was resolved using an SDS-PAGE gel and transferred to a PVDF membrane. Membranes were blocked by Sea Block blocking buffer (Pierce) for an hour at RT, followed by overnight incubation at 4 °C with the primary antibodies diluted in the blocking buffer (Table 2). Membranes were washed with 1xTBS with 0.01% Tween-20 followed by an hour incubation at room temperature with an anti-mouse Dylight 680 and anti-rabbit Dylight 800 secondary antibodies (1:2000 dilution in 1xTBST with 0.01% Tween-20). After washing the membranes with 1x TBST with 0.01% Tween-20, the membranes were imaged in Odyssey CLx imaging system (Li-COR Biosciences). All target proteins normalized to β-actin expression.

**Table 2:** List of Antibodies

Target Protein	Manufacturer	Raised in	Dilution	Application
ER-α	Santa Cruz, 543	Rabbit	1:100	ICC
Caspase-3	Cell signaling technology, 9665S	Rabbit	1:500	WB
Cleaved Caspase-3	Cell signaling technology, 9664S	Rabbit	1:500	WB
p38	Cell signaling technology, 9212S	Rabbit	1:700	WB
phospho-p38	Cell signaling technology, 4511S	Rabbit	1:700	WB
β-Actin	Santa Cruz, 58,673	Mouse	1:1000	WB
cd-31	Abcam, 28,364	Rabbit	1:50	IHC
α-Smooth muscle actin	Santa Cruz, 32,251	Mouse	1:600	IHC
VEGF	Abcam, 46,154	Rabbit	1:400	IHC
VEGFR-2	Santa Cruz, 6251	Mouse	1:400	IHC
NG2	Abcam, 129,051	Rabbit	1:100	IHC

### Chick chorioallantoic membrane (CAM) assay

Fertilized eggs were obtained from University of Urbana-Champaign Experimental Poultry facility and incubated at 38.9 °C. After 72 h, a small hole was introduced at the tip of the egg creating an air pressure that detaches the chorioallantoic membrane from the egg shell. A small window was then opened and taped and the eggs were returned to the incubator. After 96 h, a small filter paper with the treatment was put on the CAM, the window was resealed and eggs were incubated for 48 h. CAMs were harvested after fixing the membrane with 1:1 methanol: acetone (v/v) for 90 min. The membranes were viewed under a microscope and vessels sprouting around the filter paper were counted. 100% ethanol was used as the solvent for all treatments and served as the untreated control.

### TUNEL staining

TUNEL staining was performed with a DeadEnd fluorometric TUNEL system following the manufacturer’s protocol and as previously described [31].

## Statistical analysis

Statistical analysis was performed by GraphPad Prism v5.0. One-way or Two-way analysis of variance (ANOVA) was performed followed by Tukey’s range test. Statistically significant change was considered for a *p* value of < 0.05.

## Additional file

Additional file 1: **Figure S1.** ERα staining of cells BG1, HeyC2 and TOV112D cells were seeded on coverslips and fixed with ice-cold 1:1 methanol: acetone (v/v) solution for 20 min at room temperature. Following blocking for 30 min with 10% normal goat serum in 1x PBS, coverslips were incubated with an anti-ERα primary antibody for overnight at 4 °C. Following an hour of incubation with an anti-rabbit Alexa 594 secondary antibody (donkey) and 3 washes with 1x PBS, cells were mounted with DAPI Fluoromount G (Southern Biotech) and imaged using a Leica DM5500Q fluorescent confocal microscope with a Leica DFC365 FX camera. Channels were superimposed using Leica Application Suite Advanced Fluorescence version 2.6.0.7266 software. **Figure S2.** Cell viability assay after 2MeOE_2_ treatment with SB203580 MTS cell proliferation assay was performed with a CellTitre 96® Aqueous one solution cell proliferation assay kit (Promega, Madison WI) following the manufacture’s protocol. MTS reagent was treated for 4 h and absorbance was recorded at 490 nm wavelength using a BioTek Synergy HT Microplate reader. Assay performed on the three cell lines treated with 10 μM 2MeOE_2_, 10 μM SB203580 and 10 μM 2MeOE_2_ plus 10 μM SB203580 along with an untreated control group (*n* = at least 3 for each group). One-way ANOVA, error bars: SEM, *p* < 0.05. (PDF 1507 kb)

## Abbreviations

2MeOE_2_: 2-methoxyestradiol
ALA: Αlpha-linolenic acid
CAM: Chick chorioallantoic membrane
COMT: Catechol-o-methyl transferase
CYP1A1: Cytochrome p450 family 1, subfamily A, polypeptide 1
CYP1B1: Cytochrome p450 family 1, subfamily B, polypeptide 1
CYP3A4: Cytochrome p450 family 3, subfamily A, polypeptide 4
DHA: Docosahexaenoic acid
DMEM: Dulbecco’s Modified Eagle’s medium
ED: Enterodiol
EL: Enterolactone
EPA: Eicosapentaenoic acid
ER-α: Estrogen receptor alpha
MAPK: Mitogen activated protein kinase
MTS: 3-(4,5-dimethylthiazol-2-yl)-5-(3-carboxymethoxyphenyl)-2-(4-sulfophenyl)-2H-tetrazolium
PBS: Phosphate-buffered saline
SDG: Secoisolaricisresinol diglucoside
PVDF: Polyvinylidene difluoride
TBS: Tris-buffered saline
TUNEL: Terminal deoxynucleotidyl transferase dUTP nick end labeling
