# Supplementary material for: Pro-apoptotic and anti-angiogenic actions of 2-methoxyestradiol and docosahexaenoic acid, the biologically derived active compounds from flaxseed diet, in preventing ovarian cancer
Source: J Ovarian Res. 2019 May 25;12:49. doi: 10.1186/s13048-019-0523-3 (PMC6535187; doi:10.1186/s13048-019-0523-3)
Supplement: Supplementary file 1 — Figure S1. ERα staining of cells BG1, HeyC2 and TOV112D cells were seeded on coverslips and fixed with ice-cold 1:1 methanol: acetone (v/v) solution for 20 min at room temperature. Following blocking for 30 min with 10% normal goat serum in 1x PBS, coverslips were incubated with an anti-ERα primary antibody for overnight at 4 °C. Following an hour of incubation with an anti-rabbit Alexa 594 secondary antibody (donkey) and 3 washes with 1x PBS, cells were mounted with DAPI Fluoromount G (Southern Biotech) and imaged using a Leica DM5500Q fluorescent confocal microscope with a Leica DFC365 FX camera. Channels were superimposed using Leica Application Suite Advanced Fluorescence version 2.6.0.7266 software. Figure S2. Cell viability assay after 2MeOE2 treatment with SB203580 MTS cell proliferation assay was performed with a CellTitre 96® Aqueous one solution cell proliferation assay kit (Promega, Madison WI) following the manufacture’s protocol. MTS reagent was treated for 4 h and absorbance was recorded at 490 nm wavelength using a BioTek Synergy HT Microplate reader. Assay performed on the three cell lines treated with 10 μM 2MeOE2, 10 μM SB203580 and 10 μM 2MeOE2 plus 10 μM SB203580 along with an untreated control group (n = at least 3 for each group). One-way ANOVA, error bars: SEM, p < 0.05. (PDF 1507 kb) [file 13048_2019_523_MOESM1_ESM.pdf]

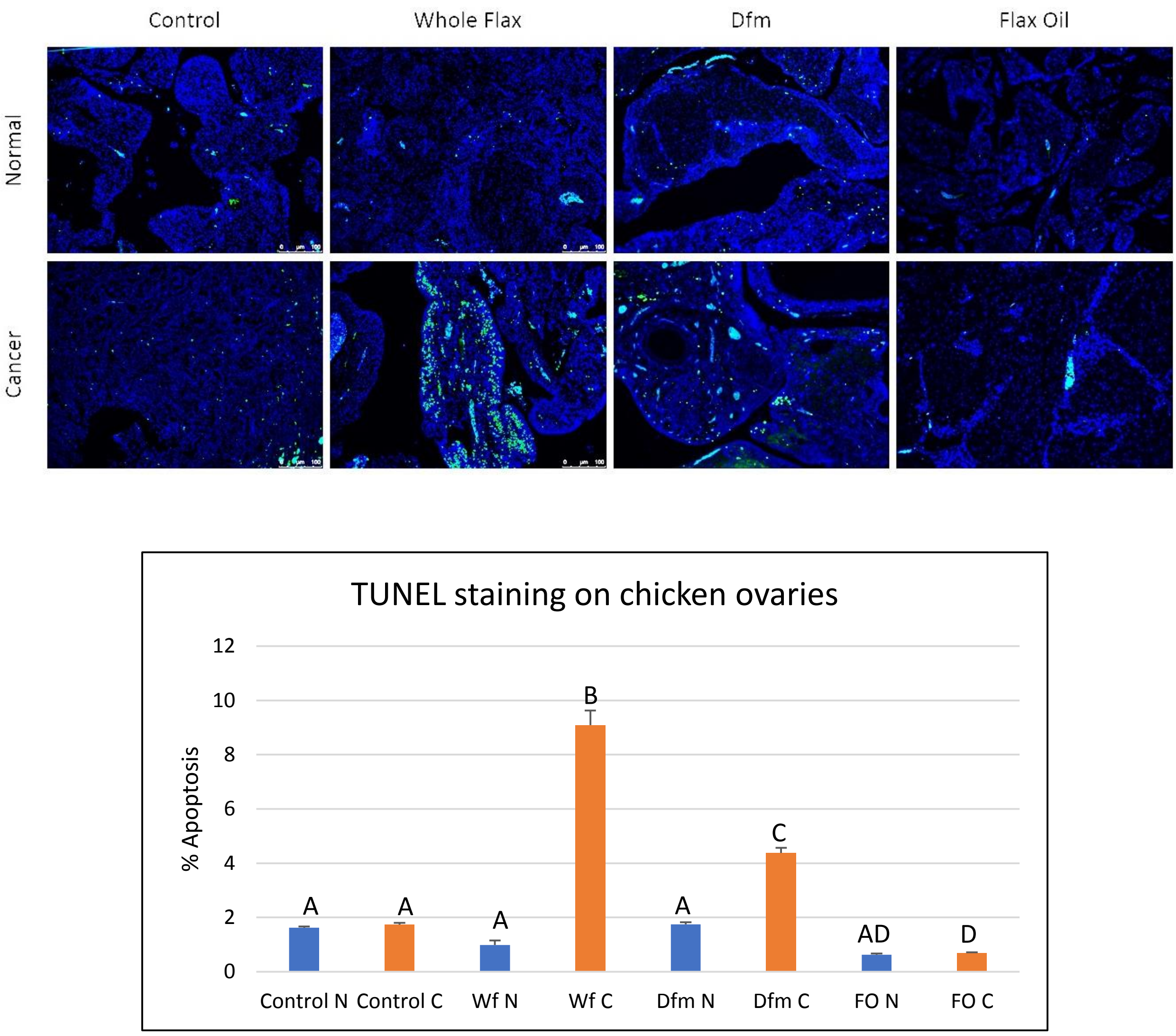
Control
Whole Flax
Dfm
Flax Oil
Normal
Cancer
TUNEL staining on chicken ovaries
% Apoptosis
12
10
8
6
4
2
0
A
A
A
B
A
C
AD
D
Control N
Control C
Wf N
Wf C
Dfm N
Dfm C
FO N
FO C

Figure 1

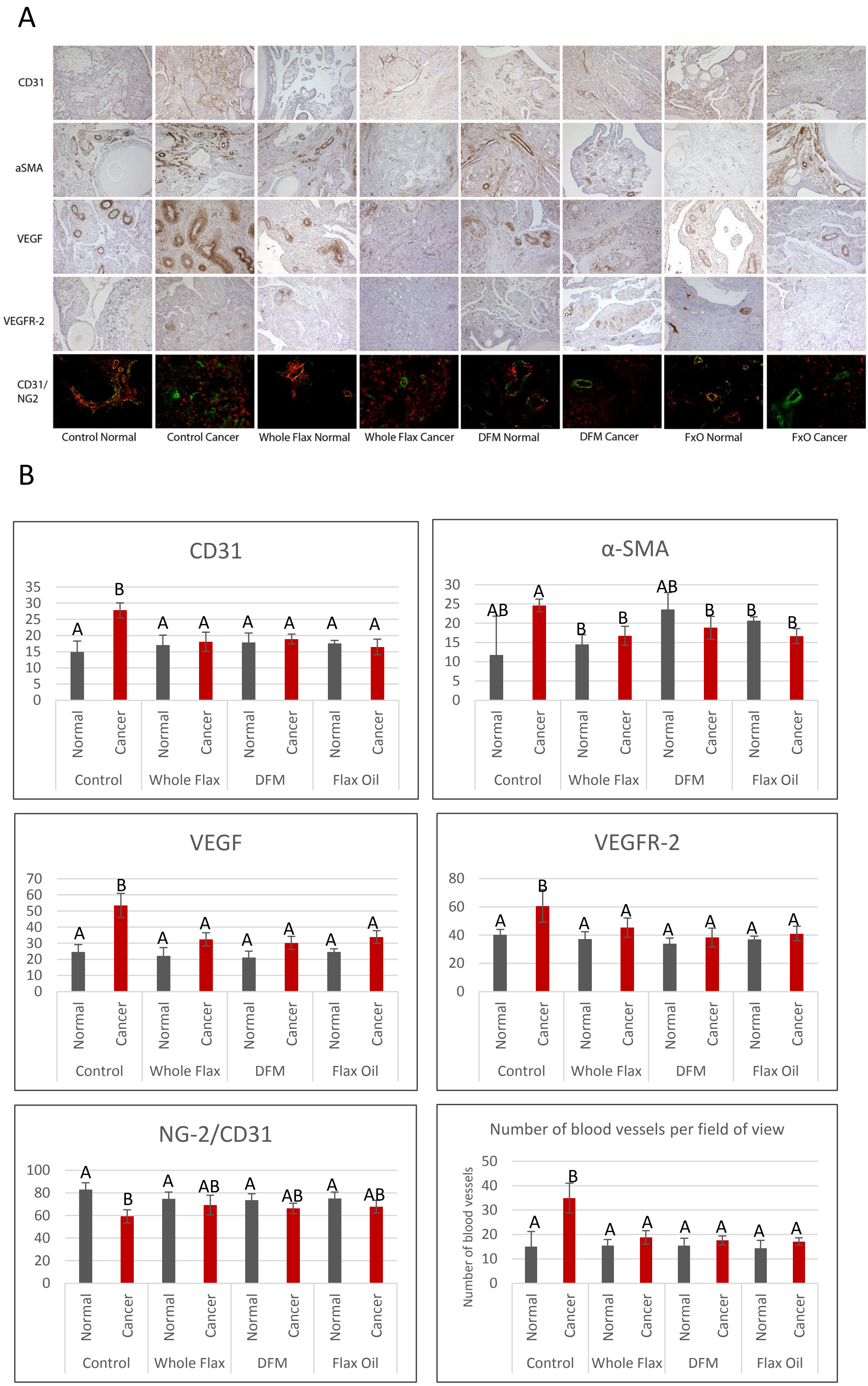
A
CD31
aSMA
VEGF
VEGFR-2
CD31/ NG2
Control Normal
Control Cancer
Whole Flax Normal
Whole Flax Cancer
DFM Normal
DFM Cancer
FxO Normal
FxO Cancer
B
CD31
α-SMA
VEGF
VEGFR-2
NG-2/CD31
Number of blood vessels per field of view
Number of blood vessels
Normal
Cancer
Control
Whole Flax
DFM
Flax Oil

Figure 2

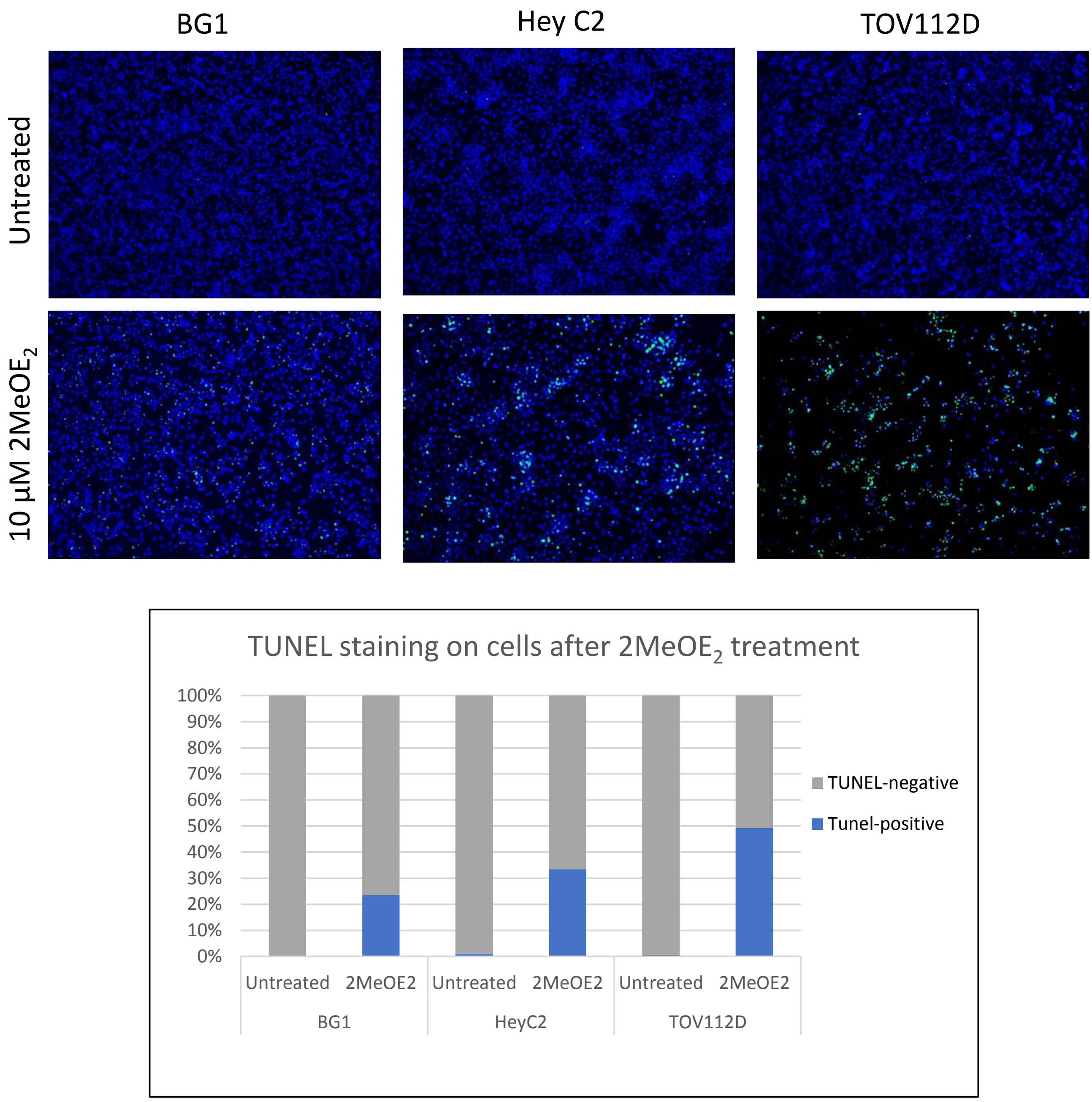
BG1
Hey C2
TOV112D
Untreated
10 µM 2MeOE2
TUNEL staining on cells after 2MeOE2 treatment
100%
90%
80%
70%
60%
50%
40%
30%
20%
10%
0%
Untreated
2MeOE2
Untreated
2MeOE2
Untreated
2MeOE2
BG1
HeyC2
TOV112D
TUNEL-negative
Tunel-positive

Figure 3

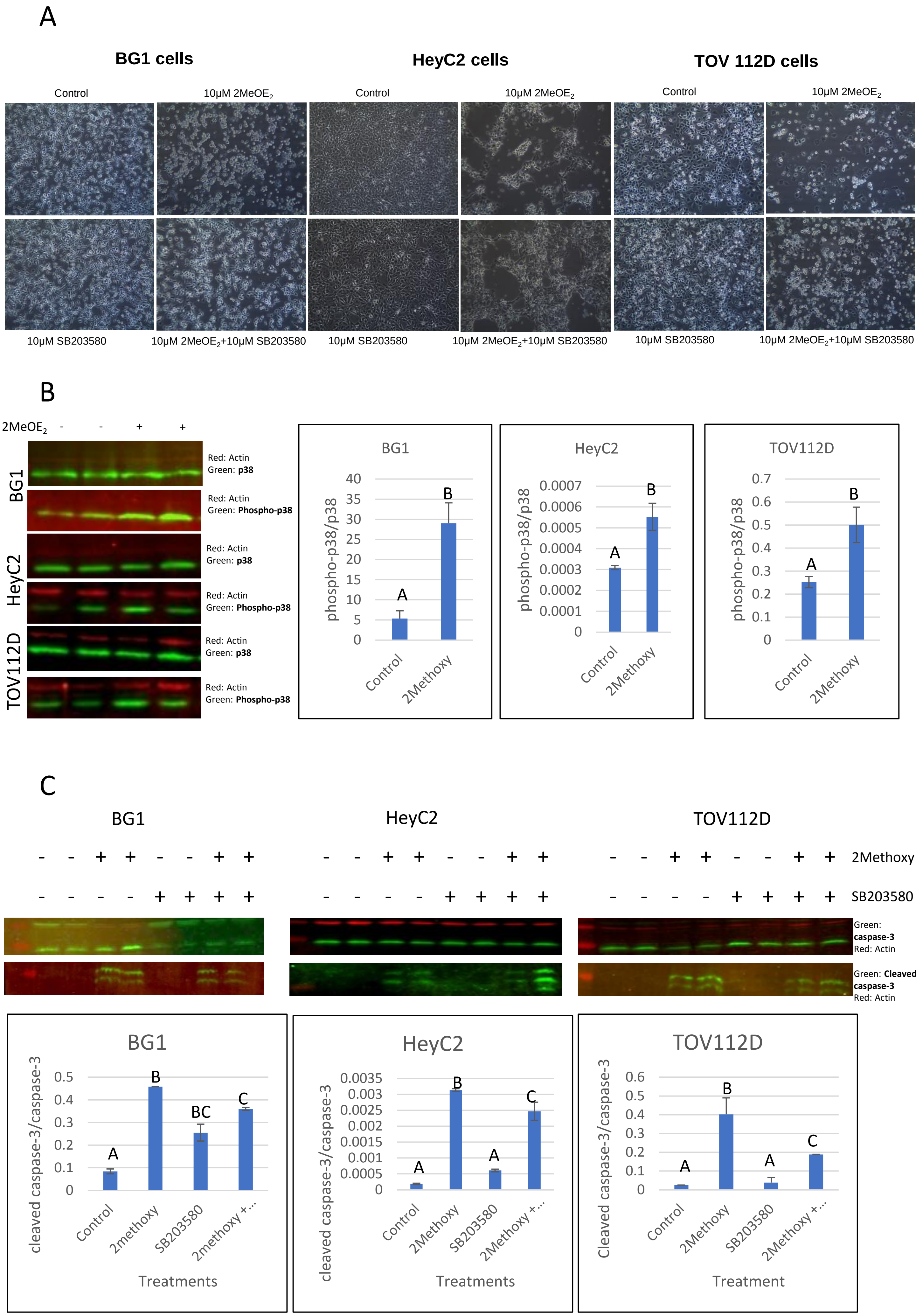
A
BG1 cells
HeyC2 cells
TOV 112D cells
Control
10μM 2MeOE2
10μM SB203580
10μM 2MeOE2+10μM SB203580
B
2MeOE2 - - + +
BG1
HeyC2
TOV112D
Red: Actin
Green: p38
Red: Actin
Green: Phospho-p38
phospho-p38/p38
Control
2Methoxy
C
BG1
HeyC2
TOV112D
2Methoxy
SB203580
Green: caspase-3
Red: Actin
Green: Cleaved caspase-3
Red: Actin
cleaved caspase-3/caspase-3
Cleaved caspase-3/caspase-3
Control
2methoxy
SB203580
2methoxy +...
2Methoxy
2Methoxy +...
Treatments
Treatment

Figure 4

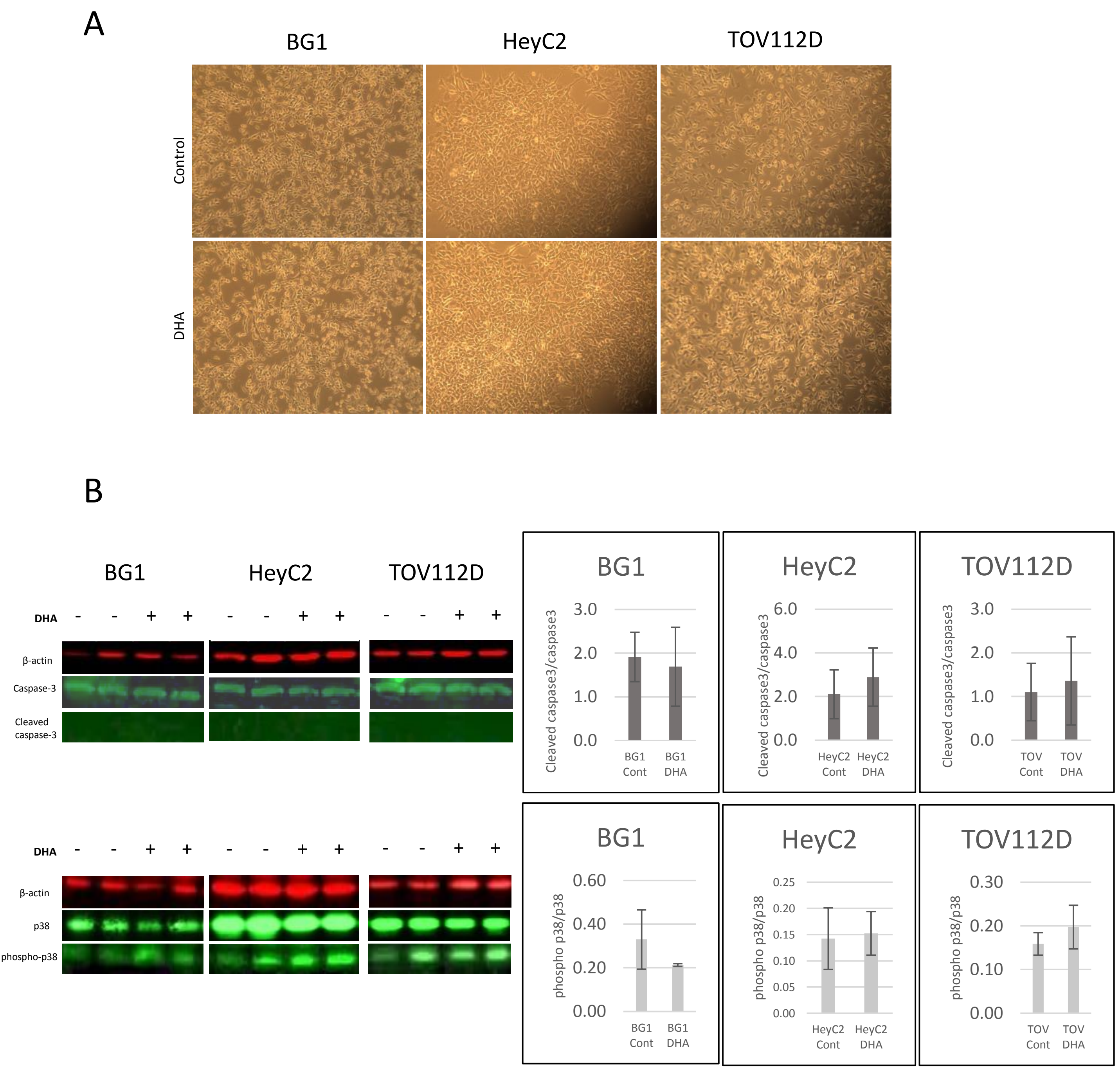
A
BG1
HeyC2
TOV112D
Control
DHA
B
BG1
HeyC2
TOV112D
DHA
β-actin
Caspase-3
Cleaved caspase-3
p38
phospho-p38
Cleaved caspase3/caspase3
phospho p38/p38
BG1 Cont
BG1 DHA
HeyC2 Cont
HeyC2 DHA
TOV Cont
TOV DHA

Figure 5

A

Control

$1\mu M\ 2MeOE_2$

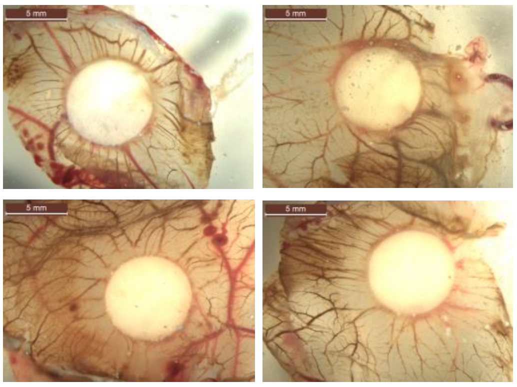

$10\mu M\ 2MeOE_2$

$100\mu M\ 2MeOE_2$

Dose dependent effect of $2MeOE_2$ on CAM

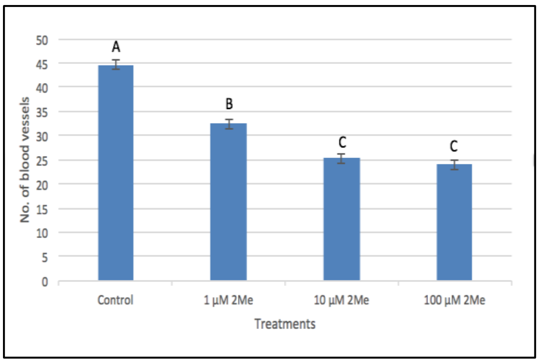

B

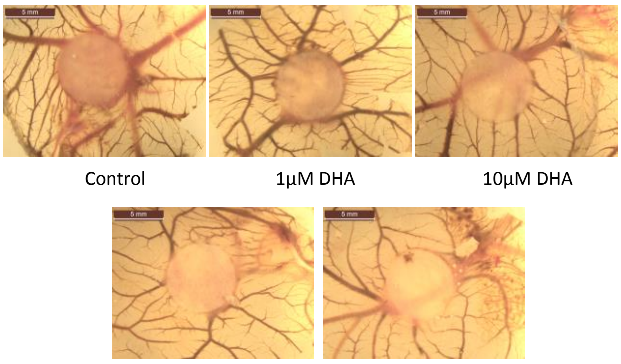

Control

1µM DHA

10µM DHA

100µM DHA

1mM DHA

Dose dependent effect of DHA on CAM

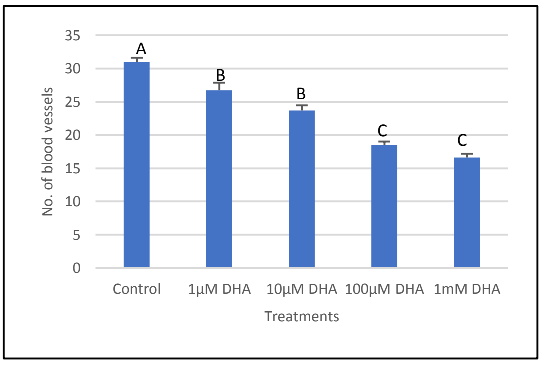

Figure 6

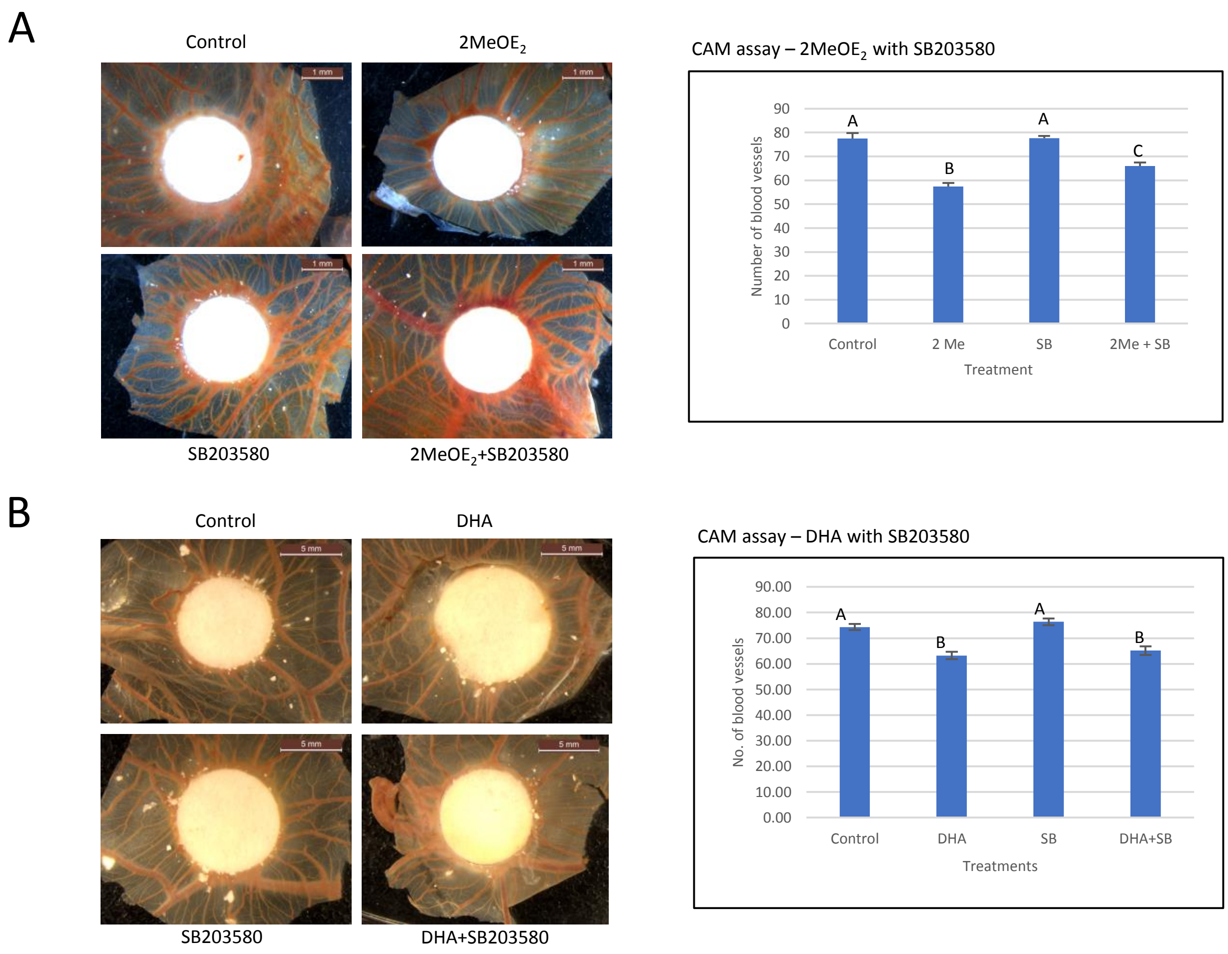
A
Control
2MeOE2
SB203580
2MeOE2+SB203580
CAM assay – 2MeOE2 with SB203580
Number of blood vessels
90
80
70
60
50
40
30
20
10
0
A
B
A
C
Control
2 Me
SB
2Me + SB
Treatment
B
Control
DHA
SB203580
DHA+SB203580
CAM assay – DHA with SB203580
No. of blood vessels
90.00
80.00
70.00
60.00
50.00
40.00
30.00
20.00
10.00
0.00
A
B
A
B
Control
DHA
SB
DHA+SB
Treatments

Figure 7

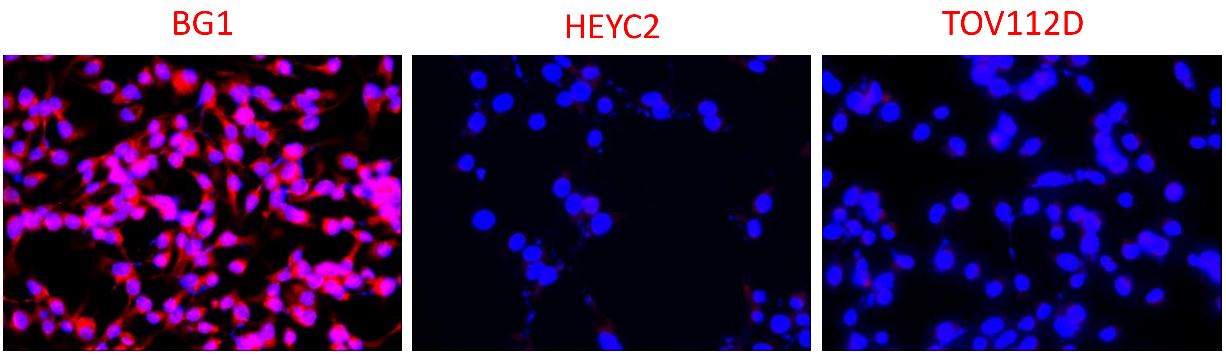

Supplementary 1

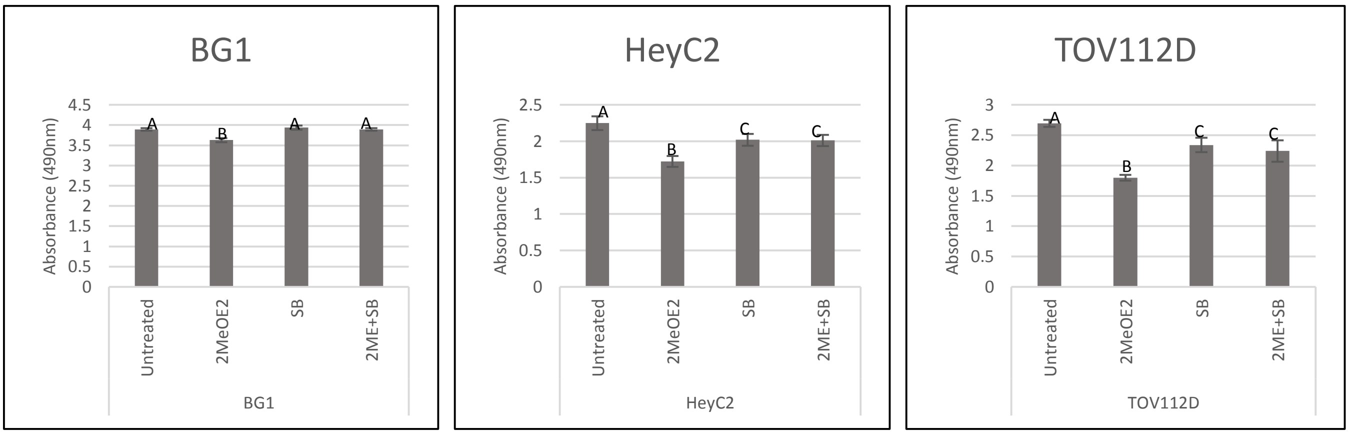

Supplementary 2
